# Chromosome Morphology and Heterochromatin Patterns in *Paspalum notatum*: Insights into Polyploid Genome Structure

**DOI:** 10.3390/genes16030242

**Published:** 2025-02-20

**Authors:** Ana I. Honfi, A. Verena Reutemann, Juan S. Schneider, Lucas M. Escobar, Eric J. Martínez, Julio R. Daviña

**Affiliations:** 1 Programa de Estudios Florísticos y Genética Vegetal, Instituto de Biología Subtropical (CONICET-UNaM), Facultad de Ciencias Exactas, Químicas y Naturales, Universidad Nacional de Misiones (FCEQyN-UNaM), Misiones 3300, Argentina; schneider.s.juan@gmail.com (J.S.S.); lucasmescobar17@gmail.com (L.M.E.); juliordavina@gmail.com (J.R.D.); 2 Laboratorio de Genética y Mejoramiento de Especies Forrajeras, Instituto de Botánica del Nordeste (CONICET-UNNE), Facultad de Ciencias Agrarias, Universidad Nacional del Nordeste (FCA-UNNE), Corrientes 3400, Argentina; vreutemann@gmail.com (A.V.R.); eric@agr.unne.edu.ar (E.J.M.)

**Keywords:** chromosome banding patterns, karyotype evolution, forage grasses, constitutive heterochromatin, polyploid, genome size

## Abstract

Background/Objectives: *Paspalum notatum* is a key multipurpose species native to American grasslands. This study provides, for the first time, a detailed karyotype analysis of diploid (2*n* = 2*x* = 20) and tetraploid (2*n* = 4*x* = 40) accessions of *P. notatum*, the most common cytotypes within the species. Methods: The constitutive heterochromatin patterns revealed using CMA-DA-DAPI staining and genome size estimations are novel contributions to the understanding of the N genome in *Paspalum*. Results: Chromosomes were small (1.1–2.3 µm), with the diploid karyotype comprising nine metacentric pairs (one bearing microsatellites on the short arms, pair 6) and one submetacentric pair. In tetraploids, the diploid karyotype was duplicated. Heterochromatin analysis revealed two CMA^++^/DAPI^−^ bands located on the short arm and satellite of chromosome 6 in diploids, while tetraploids exhibited two to three CMA^++^/DAPI^−^ and one to two CMA^++^/DAPI^0^ bands. The proportion of GC-rich heterochromatin represented 2.8 and 3.47% of the total chromosome length in diploid and tetraploid cytotypes, respectively. Genome size analysis revealed a reduction in monoploid genome size in tetraploids (1Cx = 0.678 pg) compared to diploids (1Cx = 0.71 pg), consistent with the autopolyploid origin hypothesis. Conclusions: These findings provide essential cytogenetic insights and suggest only minor structural changes in the N genome following polyploidization, which could guide future studies integrating genomic and cytogenetic maps of *P. notatum*.

## 1. Introduction

Genome studies have developed a prolific mass of data about sequences, genetic information, and several genome markers, which are essential for plant biosystematics and biotechnology. Cytological chromosome markers sometimes correlate with genetic characters (genes) and molecular and cytochemical features [1]. Chromosome numbers, shape and morphology, as well as meiotic behavior, remain essential keys for understanding genetic linkage groups and conducting in-depth genome analyses. The terms “linkage group” and “chromosome” are often interchangeable, particularly when physical maps of gene positions align with genetic information for specific chromosomes [1]. Genetic linkage maps in plants are constructed from analyses of chromosomes behavior during meiosis and alleles segregation in progenies.

Gene mapping is described using distance measures in centimorgans (cM), reflecting the recombination frequency among *loci* [1,2,3]. Recombination frequencies are critical for determining the relative position of molecular markers on genetic linkage maps and identifying homologous chromosomes [4]. Homology is also detected through chromosome morphometry and structural markers. Chromosomes, as physical representations of parts of the genome, provide species-specific markers commonly used as anchor points for chromosome maps [5,6]. Such markers, often located at centromere and satellite positions, highlight specific DNA sequences, including constitutive heterochromatin, ribosomal genes (rDNA), and AT- or GC-rich regions [7,8,9,10,11,12]. These conserved DNA sequences are now routinely employed in cytogenetic screening to construct comparative chromosome maps [4].

Chromosomes carrying highly conserved coding regions, such as multicopy tandem arrays of rRNA genes, are frequently used to represent cytogenetic chromosome maps of various species [4,7,12,13]. In *Brachypodium*, 5S rDNA sequences have been employed to identify ploidy levels, as diploid species exhibit only two copies in their somatic chromosome complements, while auto- and allotetraploids display four copies [14]. Thus, the number of 5S rDNA copies can serve as a reliable indicator of ploidy in some species [14]. In contrast, the correlation between the number of 35S rDNA copies and ploidy levels is less clear across all *Brachypodium* species [11,14].

Ploidy screening and genome–chromosome assembly via flow cytometry have also proven useful for unifying the physical and molecular domains of the genome [14,15,16]. However, these modern techniques require a classical description of karyotypes to ensure accuracy and applicability. Consequently, numerous plant cytologists advocate for a standardized karyotype nomenclature to facilitate evolutionary comparisons among taxa, regardless of the approach or technique used (genetic, genomic, or cytogenetic) [1,17,18]. Genomic data can then be anchored to their respective linkage groups through cytological marker maps and karyotypes. Moreover, the unequivocal identification of chromosomes in diploid or polyploid complements necessitates a karyotypic description grounded in the consensus nomenclature established by cytogenetic studies, thereby connecting linkage groups with karyotypic structures.

Autopolyploidy, a key intra-specific diversification mechanism, involves the presence of more than two copies of the chromosome complement within a species [19,20]. This condition results in polysomic inheritance, random pairing, and segregation of homologous chromosomes, without preferential pairing partners [21]. In autopolyploid species, karyotypes are often typically duplicated versions of the diploid karyotype [10,22,23]. Structural rearrangements or constitutive heterochromatin divergence can lead to the diploidization of homologous chromosome pairs [20], affecting inheritance patterns in some autopolyploid species [21]. Sometimes, each ploidy level has variations in asymmetry due to changes in the chromosomes’ relative sizes or the DNA volume per chromosome [24]. Cytological markers like C-banding, CMA-DA-DAPI banding, or fluorescent in situ hybridization (FISH) allow the discrimination of individual homologous chromosomes within populations or species [22,25,26,27]. CMA and DAPI bind preferentially to GC- and AT-rich sequences, respectively [28], and identify different types of constitutive heterochromatin (Het-C) differing in their DNA-base composition [7,9,29].

*Paspalum* is a genus that includes species of significant economic value, primarily as forage and lawn grass, with one also serving as a minor cereal (*P. scrobiculatum*, kodo millet). *Paspalum notatum* Flüggé, known as bahiagrass, is a subtropical forage grass with diploid (2*n* = 2*x* = 20) and conspecific polyploid (2*n* = 4*x* = 40) cytotypes. It is widely distributed across native grasslands in the Americas, from Mexico to Argentina [30,31,32]. This species, significant for forage production in the subtropical belt worldwide, particularly in Southeastern USA, belongs to a polymorphic agamic polyploid complex [31]. Diploid sexual biotypes are geographically restricted, while apomictic polyploids are more widespread [31,33,34,35]. It is a common species in American rangelands, particularly in South America, where it forms a resilient tapestry that withstands trampling, grazing, and environmental changes. Additionally, it is the third most important species of the genus in terms of the number of released cultivars [31]. The diploid cytotype is commonly referred to as Pensacola bahiagrass and there are derived hybrids, released and cultivated for livestock food [31].

Two taxonomic varieties differentiated by their morphology and ploidy levels are the most frequent biotypes in nature. *Paspalum notatum* Flüggé var. *saurae* Parodi is a self-incompatible sexual diploid (2*n* = 2*x*= 20) biotype [33,34,36]. *Paspalum notatum* var. *notatum* is a tetraploid (2*n* = 4*x*= 40) biotype, which is apomictic, pseudogamous, and self-compatible [35]. The tetraploid variety is the most common biotype in nature [35]. Rare triploid and pentaploid individuals have occasionally been collected in natural populations [37,38].

The intra-specific homology between diploid and tetraploid chromosomes has been observed in hybrids from heteroploid crosses, which exhibited homologous pairing during meiosis, sharing the same genome (N genome) in *Paspalum* [39,40,41]. Numerous efforts have been made to understand the genetic mechanisms of apomixis in *P. notatum*. These include the identification of apomixis inheritance, the apomixis *locus* (region), molecular markers linked to apospory (type of gametophytic apomixis), syntenic genetic comparison with other species, and transcriptomic and genomic sequencing of linkage groups [32,33,34,42,43]. Previous studies have shown that apomixis (apospory type) in bahiagrass is controlled by a single dominant gene with a distorted segregation ratio [33,34].

A genetic linkage map constructed from analyses of segregating progenies in diploid and tetraploid cytotypes evidenced a single linkage group associated with apospory, showing recombination restriction and preferential chromosome pairing [43,44,45]. Additionally, polysomic and disomic inheritance has been observed in tetraploids of *P. notatum*, with preferential chromosome pairing (disomic inheritance) detected in the chromosome segment related to apospory [42]. More recently, a high-resolution genome assembly at the chromosome scale for the diploid cytotype of *P. notatum* was developed using Oxford Nanopore Technology (ONT), identifying eight of the ten chromosome centromeres in detail [46,47]. Diverse repetitive elements and gene densities were assigned to each chromosome pair in the diploid *P. notatum* genome [47]. Despite these advances, the karyotype and constitutive heterochromatin distribution of *P. notatum* cytotypes have not yet been described.

This study aimed to (i) describe the karyotype using chromosome morphometry of the two most common biotypes (cytotypes 2*x* and 4*x*) of *P. notatum*; (ii) analyze the constitutive heterochromatin distribution in diploid and tetraploid biotypes of bahiagrass; (iii) estimate the genome size of each cytotype; and (iv) contribute to an integrative and comprehensive cytogenomic description of *P. notatum*.

## 2. Materials and Methods

### 2.1. Plant Material

Five accessions of *P. notatum* were collected from different localities (Table 1). Voucher specimens were deposited in the Herbarium of the Universidad Nacional de Misiones (MNES), Argentina. Rhizome cuttings were cultivated in the greenhouse of the Instituto de Biología Subtropical (IBS, UNaM-CONICET) and the Botanical Garden A. Roth in Posadas (Misiones, Argentina) as part of the germplasm collection. Pieces of rhizomes were grown in pots with professional substrate to obtain root-tip meristems for chromosome preparations.

### 2.2. Chromosome Preparation

Root-tip meristems obtained from young root tips (1–1.5 cm long) were pretreated with a saturated solution of 1-alphabromonaphtalene in water for 3 h at 24 °C and then fixed in ethanol/glacial acetic acid (3:1) for at least 24 h at 4 °C. Fixed root-tips were stored in the fixative at 4 °C until used.

### 2.3. Feulgen Staining and Karyotype Analyses

Root-tips were hydrolyzed in 1N HCl at 60 °C for 10 min, stained with Schiff’s reagent (Basic Fuchsine, BA, BERNA, Buenos Aires, Argentina) for 3 h in darkness, and disaggregated over a drop of 2% acetic orcein before squashing. Semi-permanent slides were prepared by sealing the coverslip with rubber solution to examine chromosome number and morphology.

### 2.4. Karyotype Morphometry

Chromosome morphometric parameters were averaged from measurements of at least 10 optimal mitotic metaphases. For karyotype analyses, at least 10 well-spread chromosome plates per specimen were chosen. The nomenclature of Levan et al. [48] to describe chromosome morphology, centromere position, and type was applied. Karyotype parameters were determined using Micromeasure 3.3 [49]. Photographs were used to measure features of each chromosome pair, including short arm length (*s*), long arm length (*l*), and total chromosome length (*c = s + l*). The length of the satellite (when observed) was added to the respective chromosome arm [48].

The following morphometric parameters were calculated: total chromosome length of the complement (TCL *=* ∑c), centromeric index (*i* = 100 × *s*/*c*), and arm ratio (*r* = l/*s*). Chromosome pairs were arranged by type and decreasing size to draw idiograms [48]. Chromosomes were classified into two categories: metacentric (m, *i* = 50–37.5) and submetacentric (sm, *i* = 37.5–25). Relative chromosome length was estimated as haploid total chromosome length (HTCL) in diploids and as a percentage of TCL in tetraploids.

### 2.5. Idiograms Construction

A basic idiogram was constructed using mean values for the diploid cytotype to represent diagrammatically the chromosome complement (karyotype) of the species. An idiogram defines chromosome number and morphology and is species-specific [9,50]. The idiogram was prepared using AutoCad v2006. Satellites were classified following Battaglia’s nomenclature [51,52]. Similar measurements were performed in chromosome banding, including the size and position of heterochromatic blocks.

### 2.6. Karyotype Asymmetry

Karyotype asymmetry was analyzed using Stebbins‘s categories [53] and Romero Zarco [54] intra- and inter-karyotypic asymmetry indexes (A_1_ and A_2_). The A_1_ index was used to measure intra-chromosomal asymmetry, applying the following formula: A_1_ = 1 − [(Ʃ*si*/*li*)/*n*], where *si* and *li* are the average lengths of the short and long arms of each chromosome, respectively, and *n* is the number of homologous chromosomes. The A_1_ index varies from 0 to 1 and approaches 0 when chromosomes tend to be metacentric [54]. The A_2_ index was estimated using the relationship between the standard deviation and the mean of the length of the chromosomes of each cytotype, where A_2_ = *S*/x*c*, where *S* corresponds to the standard deviation and x*c* is the mean of the length of all the chromosomes of the complement [54]. The ratio of the shortest to the longest chromosome pair (*R* = *cMax*/*cMin*) and the *arm ratio* (*r*) were used to determine asymmetry [53]. These indexes indicate heterogeneity in chromosome length and centromere position among cytotypes.

### 2.7. Chromosome Preparation for Constitutive Heterochromatin Detection

For molecular cytogenetical analysis, the protocols of Schwarzacher et al. [55] and Daviña [56] were applied with minor modifications. Pre-treated root meristems were initially washed in distilled water and then rinsed in citrate buffer (0.01 M, pH 4.8) for 5 min. The tissues were subsequently digested in an enzyme solution containing 2% cellulase (*w*/*v*, Cellulase Onozuka R-10, from *Trichoderma viride*, PhytoTechnology laboratories C224, St Lenexa, KS, USA) and 20% pectinase (*v*/*v*, Pectinase Solution from *Aspergillus niger* Sigma Alldrich, P4716-25ku Merck Group, St. Louis, MO, USA) in citrate buffer for approximately 3 h at 37 °C. After enzymatic digestion, the tissues were rinsed again in citrate buffer, macerated in 45% acetic acid, and squashed to prepare the slides.

Slides containing well-spread mitotic metaphase plates were selected for further analysis. To remove the coverslips, the slides were briefly exposed to liquid nitrogen (−196 °C) and then air-dried at room temperature for two days. The prepared slides were stored at −20 °C until they were ready for use in subsequent analyses.

### 2.8. Chromosome Sequential Tri-Staining with Fluorochromes CMA/DA/DAPI

The Schweizer [57] protocol, with modifications, was used for staining with CMA/DA/DAPI (chromomicyn A_3_, from *Streptomyces griseus*, Sigma Alldrich, C2659, Merck Group, St. Louis, MO, USA; distamicyn A from *Streptomyces distallicus* Sigma Alldrich, D6135 Merck Group, St. Louis, MO, USA and 4′-6-diamidino-2-phenilindol, Sigma Alldrich, D9542 Merck Group, St. Louis, MO, USA). The constitutive heterochromatin is revealed by these fluorochrome stains as bands which will be named CMA^+^, CMA^++^, or CMA^−^ (indicating presence or absence of CG-rich DNA sequences) and DAPI^+^, DAPI^−^ (indicating AT-rich or AT-poor DNA sequences), or DAPI^0^ (neutral bands whose composition is not clearly understood [29]). The combination of CMA^+^/DAPI^0^, i.e., chromomycin bright and DAPI indifferent, indicate moderately GC-rich DNA. CMA^++^ indicates strongly brilliant. Slides were aged for two days, then stained with CMA (10 µL of solution: McIlvaine buffer, pH 7, 10 mM MgCl_2_, 0.12 mg/mL CMA) for 1 h at room temperature. After rinsing and drying, slides were treated with DA (1 µg/mL in McIlvaine buffer) for 15–30 min, rinsed, and air-dried. Staining with DAPI (1–2 µg/mL) followed for 30–45 min in darkness. Slides were mounted in glycerol/McIlvaine buffer (1:1) with 5 mM MgCl_2_ and aged for 3–5 days before analysis.

Chromosomes were observed using a Leica DML epifluorescence microscope and photographs were taken with a DF C310 FX video equipment using Leica LAS V4.0 software. Measures of stained chromosome were made using the software Micromeasure 3.3 [49] to identify each of them. Later, microphotographs were edited in Adobe Photoshop version 7.0. The constitutive heterochromatic regions in chromosomes were determined based on bands revealed using the three base-specific fluorochromes (DAPI/DA/CMA), where chromomycin A3 (CMA_3_) exhibits the distribution of GC-rich heterochromatin sequences and DAPI reveals AT-rich heterochromatin [7]. The signal sizes were measured along each banded chromosome and then expressed as a percentage of the total length of the chromosome complement.

### 2.9. Genome Size Measurements

Genome size was estimated using the CyStain PI absolute P kit (Partec, Görlitz, Germany) and measured with a CyFlow^®^ Space flow cytometer (Sysmex-Partec, Görlitz, Germany). Leaf nuclei were isolated using a Tissuelyser, followed by staining with propidium iodide (PI) and RNase. DNA content was calculated by comparing the fluorescence of samples and standards. *Zea mays* CE-777 (5.43 pg) was used as the internal standard for diploids, which has 5.43 pg and 1Cx = 2.655 Mbp. For the tetraploids, *P. notatum* H1961 (1.438 pg) was used as the internal standard. The mean of three repetitions was used for each accession, with 5000–10,000 nuclei analyzed per sample. To convert DNA mass to base pairs, the formula 1 pg = 0.978 × 10^9^ bp was applied [57,58].

## 3. Results

### 3.1. Karyotype of P. notatum

**Figure 1:**
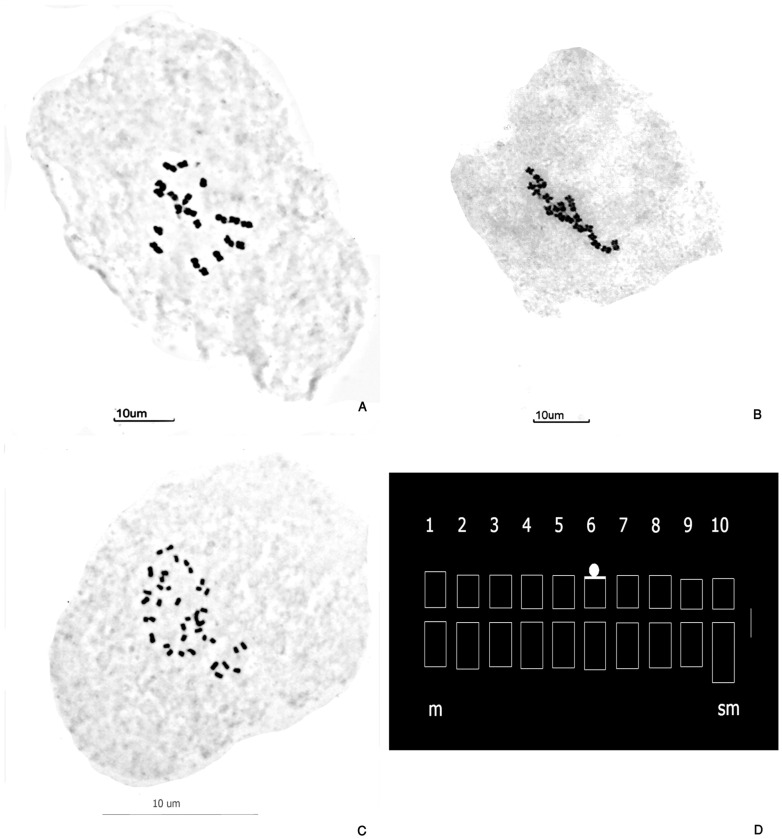
Mitotic metaphases of *P. notatum* stained using Feulgen technique. (**A**) Diploid cytotype with 2*n* = 2*x* = 20, NN chromosomes (H1453 #1). (**B**) Diploid accession H1453 #2. (**C**) Autotetraploid cytotype with 2*n* = 4*x* = 40, NNNN chromosomes (H1603 #1). (**D**) Idiogram of the basic (haploid) karyotype (9 *m* + 1 *sm*) of N genome of *P. notatum* (2*n* = 2*x* = 20, and 2*n* = 4*x* = 40). Constitutive heterochromatin (GC-rich regions) is indicated with white shading. Note the microsatellite in the short arm of pair 6. Chromosome types: metacentric (*m*), submetacentric (*sm*), as classified according to Levan et al. [48] (1964). (**A**–**C**) Scale bar = 10 µm. (**D**) Scale bar = 1 µm.

All diploid and tetraploid plants consistently exhibited 20 and 40 chromosomes, respectively (Table 1, Figure 1A–C).

The karyotype of *P. notatum* var. *saurae* consisted of 16 metacentric (*m*) and 4 submetacentric (*sm*) chromosomes, with total chromosome lengths (*c*) ranging from 1.3 to 2.3 µm (Table 2). The total chromosome length (TCL) of the diploid cytotype was 34.28 µm. The longest chromosomes were the metacentric pair 1, with a mean length of 2.209 ± 0.16 µm, followed by the submetacentric pair 10, with a mean length of 1.94 ± 0.158 µm. The smallest chromosome pair was the metacentric pair 9, measuring 1.327 ± 0.103 µm (Table 2). Little differentiation among the metacentric pairs was observed. Secondary constrictions were present in a single chromosome pair (pair 6), which also featured a microsatellite located at the distal position of the short arm (Table 2, Figure 1D). The microsatellites were not regularly observed in all cells.

The karyotype of *P. notatum* var. *notatum* consisted of 36 *m* and 4 *sm* chromosomes, with a *c* ranging from 1.1 to 2.4 µm (Table 3). The tetraploid cytotype showed a quadruplication of the haploid karyotypic formula (9 *m* + 1 *sm*) (Table 3, Figure 1D). The TCL of tetraploids was 63.94 µm. The TCL of the tetraploid cytotype was not a direct duplication of the diploid TCL (34.28 µm) (Table 4). The longest chromosome quartets in the tetraploid complement corresponded to the metacentric quartet 1 and the submetacentric quartet 10 (Table 3). Similarly, the smallest chromosome lengths were in the metacentric quartet 9 (Table 3). The distal microsatellite on the short arm of the quartet 6 was consistently maintained (Table 3 and Table 4).

The basic karyotype formula was shared between the ploidy levels and represented in a consensus idiogram (Figure 1D). The karyotypes of diploid and tetraploid cytotypes displayed remarkable similarity and preserved key structural features (Table 4).

### 3.2. Constitutive Heterochromatin in Paspalum notatum

**Figure 2:**
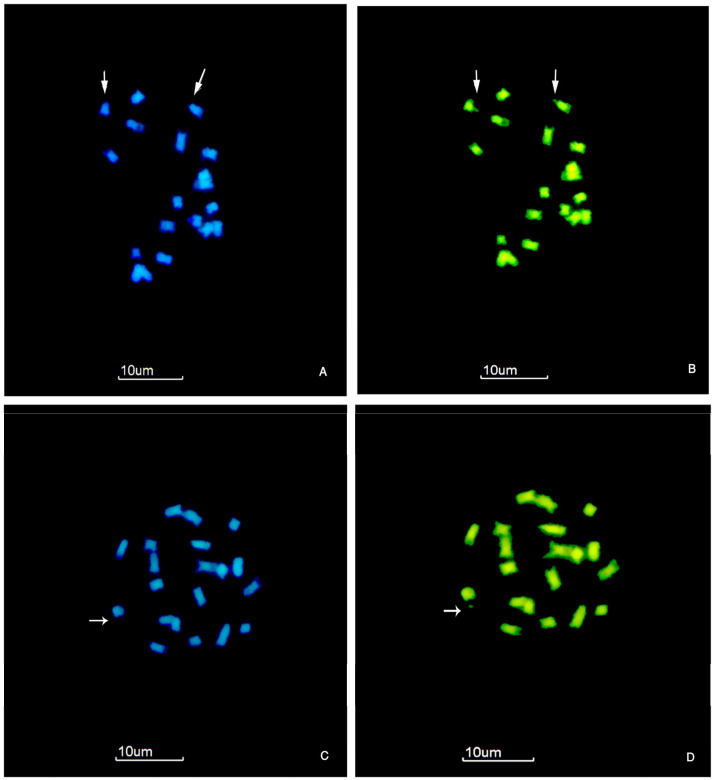
Triple sequential staining with CMA/DA/DAPI of mitotic chromosomes of diploid *P. notatum* (2*n* = 2*x* = 20). (**A**) Cell showing two chromosomes with DAPI^−^ satellites (white arrows). (**B**) The same cell as in (**A**) with CMA^+^ satellites (white arrows). (**C**) Somatic cell displaying a DAPI^−^ chromosome band and satellite. (**D**) The same cell as in (**C**) with CMA^+^ chromosome band and microsatellite. CMA staining: bright green; DAPI staining: blue. Accession H1453 #1. Scale bar = 10 µm.

**Figure 3:**
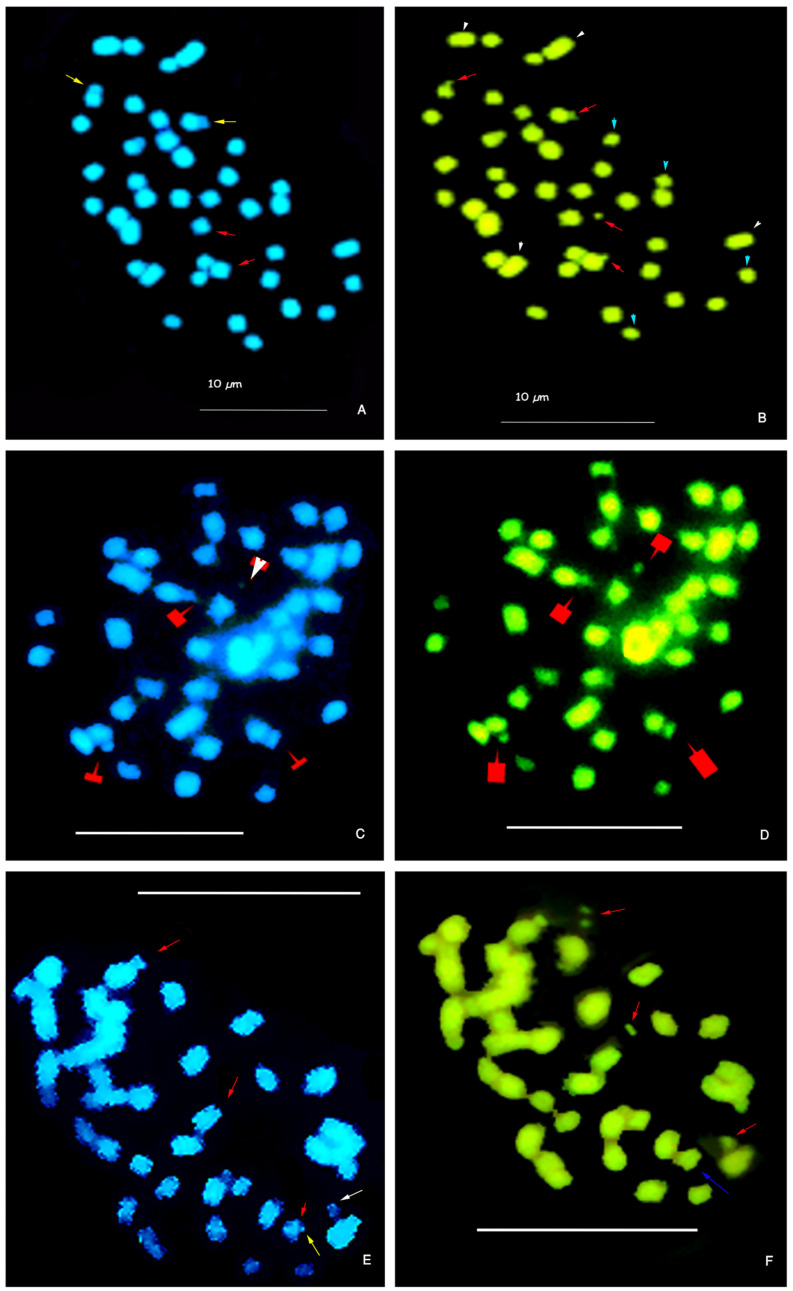
Triple sequential staining with CMA/DA/DAPI of mitotic chromosomes of tetraploid *P. notatum* (2*n* = 4*x* = 40). (**A**) Somatic cell with four microsatellites marked: two DAPI^−^ (red arrows) and two DAPI^0^ (yellow arrows). (**B**) The same cell as in (**A**) showing the four CMA^+^ satellites (red arrows). The red arrows indicate the chromosomes of quartet 6, formed by chromosomes carrying a microsatellite on a short arm. The white arrowheads indicate the chromosomes from the quartet 10, and the blue arrowheads indicate the quartet 9. (**C**) Somatic cell with DAPI staining: three microsatellites are DAPI^0^ (red arrows indicate faintly colored neutral bands) and one is DAPI^−^ (white arrowhead indicates the place where the DAPI band is absent). (**D**) The same cell as in (**C**) with four CMA+ satellites (red arrows). (**E**) Somatic cell showing three DAPI^−^ satellites (red arrows) and one DAPI^0^ satellite (white arrow). Note the absence of the distal end of the short arm of the chromosome carrying the DAPI^0^ satellite (yellow arrow). (**F**) The same cell as in (**E**) with three clear CMA^+^ satellites (red arrows) and a CMA^+^ band on a short arm (blue arrow). Note the chromosome lacking a visible microsatellite (blue arrow). CMA staining: bright green and DAPI: blue. Accession H1603 #1, #2. Scale bar = 10 µm.

Predominantly, CMA^++^/DAPI^−^ bands were observed (Figure 2 and Figure 3). In the diploid cytotype, the metacentric pair 6 carries a microsatellite on the distal short arm (Figure 1D and Figure 2B,D). The CMA^++^/DAPI^−^ bands covering this region and the microsatellite indicate a GC-rich composition (Figure 2A–D, Table 4). In some cells, only one CMA^++^/DAPI^−^ microsatellite is visible instead of two (Figure 2C,D). The proportion of Het-C reached in the diploid cytotype reached 2.8% of the TCL (Table 4 and Table 5).

In the tetraploid cytotype, a GC-rich band (CMA^++^/DAPI^−^) of approximately 0.5 µm was detected, covering a small segment of the distal short arm and the microsatellite in the metacentric quartet 6 (Figure 3A,B).

The chromosomes in quartet 6 exhibited varying banding patterns. The most common pattern included four bands: two CMA^++^/DAPI^−^ and two CMA^++^/DAPI^0^ bands (Figure 3A,B). A less frequent pattern showed three CMA^++^/DAPI^–^ bands and one CMA^++^/DAPI^0^ band (Figure 3B,C). These bands were located at the secondary constriction and microsatellites. Occasionally, one microsatellite was not clearly visible, and one of the CMA^++^/DAPI^–^ bands displayed reduced DAPI staining due to the overlapping of the chromosome arm (Figure 3E,F).

All microsatellites had Het-C bands with CMA^+^ (GC-rich regions), but the number of microsatellites with CMA^++^/DAPI^0^ varied per cell. Microsatellites with DAPI^0^ signals exhibited faint staining and were typically found at two (Figure 3A,B) or one (Figure 3C–F) per cell. No cells displayed all four bands as CMA^++^/DAPI^−^ or CMA^++^/DAPI^0^. The amount of Het-C in the tetraploid cytotype was 3.47%, and the increase in TCL was not associated with a proportional increase in Het-C or the expected duplication from the diploid karyotype (Table 4 and Table 5).

### 3.3. Genome Size of P. notatum

The diploid cytotype exhibited a genome size of 1.41 ± 0.008 pg, while the tetraploid cytotype had a genome size of 2.753 ± 0.015 pg (Table 6). This is the first report of the absolute DNA content (C-DNA_abs_) for diploid and tetraploid *P. notatum*. The monoploid genome size (1Cx) was 0.713 pg in the diploid cytotype and 0.678 pg in the tetraploid cytotype (Table 6). Genome downsizing in tetraploids accounted for a reduction of 0.035 pg or approximately 0.03423 × 10^9^ bp.

Furthermore, the genomic size of both cytotypes differed by 4.62 µm in length (TCL, Table 4). The TCLs indicated that the duplication of the chromosome complement was not proportional to ploidy levels (TCL_2x_ = 34.28 µm vs. TCL_4x_ = 63.94 µm) (Table 4).

## 4. Discussion

### 4.1. Chromosome Evidence of Remodeling and Downsizing of N Genome of Paspalum notatum

Cytogeographically, *P. notatum* germplasm from America has been extensively analyzed, and in line with these findings, all studied accessions exhibited chromosome numbers consistent with previous reports for the species [35,60,61,62,63]. The diploid biotype of bahiagrass is rare in nature, with most accessions originating from Santa Fe, Argentina [35,47,63]. In this study, we report two new diploid populations from Entre Ríos, Argentina, and Itapúa, Paraguay, suggesting long-distance dispersal beyond the typical distribution range of this cytotype.

The shared karyotype formula of diploid and tetraploid *P. notatum* confirms the autopolyploid origin of this polyploid complex. Here, we propose a basic karyotype formula for the N genome of *Paspalum* constituted by nine metacentric and one submetacentric chromosomes (9 *m* and 1 *sm*), corresponding to 1Cx DNA content. Furthermore, cytotypes of *P. notatum* shared the karyotype formulae independently of the conspicuous morphological differences among taxonomic varieties. Reutemann et al. [10] observed similar results in the diploid–autotetraploid complex of *Paspalum cromyorhizon* Trin. ex Döll, in which both cytotypes shared the karyotype formulae. *Paspalum notatum* and *P. cromyorhizon* share the N genome of *Paspalum* [41].

The N genome is also present in other species, such as *P. pumilum* Nees, *P. equitans* Mez, and *P. ionanthum* Chase [10,41,64,65,66,67,68]. All these species belong to the informal taxonomic group *Notata* [69]. Notata species exhibit four alternative genetic systems [70]. For all Notata genetic systems, we expect the same karyotype formula, regardless of the ploidy or reproductive behavior (selfing, outcrossing, or apomictic). Nevertheless, we observed that the mean chromosome length (*c*) in the tetraploid cytotype of *P. notatum* was smaller than the diploid cytotype, likely due to genome adjustment during post-polyploidization events. Karyotype asymmetry in the tetraploid complement suggested minor changes in the DNA content per chromosome without altering the chromosome morphology. Differential patterns of DNA content among chromosomes or arms, even in the absence of chromosomal rearrangements, could lead to asymmetry changes in the karyotype [71]. Minor-size chromosome changes may have occurred during the process of autopolyploidization in *P. notatum*, as tetraploids are not an exact duplicate of the diploid intra-specific counterpart.

Recent works depicting karyotypes have often used the total chromosome length (*TLC*), omitting chromosome morphology based on the centromeric index (*i*) or disregarding the decreasing length order within each chromosome morphotype [48]. For example, in *P. quadrifarium* Lam., prometaphase chromosomes were used for karyotype analyses of triploid (2*n* = 3*x* = 30) and tetraploid (2*n* = 4*x* = 40) accessions, demonstrating segmental allopolyploidy [72]. However, the chromosome arrangement within ten groups of three and four chromosomes, respectively, was based on heteropyknosis of the prometaphase chromosome length [72], making this approach incompatible with a comparative analysis of karyotype evolution in the genus. Additionally, the lack of karyotype studies in *Paspalum* species is often attributed to the small size of chromosomes and widespread polyploidy. In general, *Paspalum* chromosomes are small (1–3 µm) and vary in number from 2*n* = 20 to 160 ([73] and references therein). The absence of consensus karyotypes in the genus could lead to several ambiguous chromosome frameworks for comparative genome studies.

### 4.2. Genome Evidence of Remodeling and Downsizing of N Genome of P. notatum Polyploid Complex

The constitutive heterochromatin (Het-C) in *P. notatum* consisted of repetitive GC-rich sequences of DNA and exhibited conserved block localization associated with chromosomes carrying microsatellites (SAT-chromosome). Additionally, the same linkage group (pair 6 in diploids and quartet 6 in tetraploids) retained the SAT- and GC-rich Het-C associated with secondary constrictions. The Het-C of the metacentric quartet 6 in the tetraploid cytotype varied in its base composition. Specifically, one of the chromosomes had a GC-rich Het-C band in the distal position of the short arm (CMA^++^/DAPI^−^), which was absent in the other three chromosomes of the quartet 6.

The increased number of Het-C bands in tetraploid *P. notatum* is attributable to chromosome duplication. Similarities in heterochromatin patterns between tetraploid and diploid individuals provide additional evidence for an autopolyploid origin of *P. notatum*. Although the tetraploid cytotype had more constitutive heterochromatin (Het-C_2x_ = 2.8% per TCL; Het-C_4x_ = 3.47% per TCL), it was not proportional to the expected duplication of the diploid amount (Het-C_4x_ expected = 5.6% per TCL). This suggests that the tetraploid cytotype underwent small structural changes, remodeling the genome without altering the chromosome morphology or karyotype constitution. Furthermore, this disproportionate duplication was also observed for TLC.

In *P. notatum*, karyotype asymmetry was increased in tetraploids. However, the Het-C banding pattern exhibited identical position across ploidy levels, particularly along the subtelomeric regions of short arms and microsatellites. The preferential localization of Het-C on SAT chromosomes is common in angiosperms [7,8]. This aligns with the predominant CMA^+^ and DAPI^−^ banding pattern for heterochromatin associated with secondary constriction and nucleolar organizer regions (NORs-Het-C) [7].

The presence of variable CMA^++^/DAPI^0^ bands in the tetraploid cytotype of *P. notatum* is atypical for a region where an NOR localization is expected. This staining pattern could be associated with functional variation in the region, as proposed for Ag-NOR (silver stained NORs) banding, which reveals sites that are transcriptionally active or potentially active during the preceding interphase [28,74]. Further analyses with Ag-NOR banding and 45S–35S FISH could confirm this assumption. Similarly, in diploid species of the Quadrifaria group of *Paspalum* (*P. haumanii* Parodi, *P. intermedium* Munro ex Morong and Britton, *P. quadrifarium* Lam.), Het-C banding revealed that the SAT chromosome was submetacentric, and satellites were CMA^++^/DAPI^−^, whereas no secondary constrictions were detected in tetraploids [75]. Furthermore, FISH experiments in diploid and tetraploid species have demonstrated variable localization and numbers of 45S and 35S rDNA sites independent of ploidy [75,76]. Most comparisons between species analyzed by Vaio et al. [75] were inconsistent due to the use of prometaphasic chromosomes with incomplete condensation, unlike in this study.

A comparison of Het-C band patterns among species revealed that the Het-C distribution across the genome is not homogeneous [7,28,77,78]. Het-C varies qualitatively and quantitatively between species, exhibiting polymorphism in the number and size of bands, base composition, and distribution [7,28,77,78]. In the diploid–tetraploid system of *P. notatum*, the presence and proportion of Het-C bands relative to the TCL and nuclear DNA content varied, but the number of chromosomes carrying bands was consistent with intra-specific ploidy level. A similar case was reported for *Cynodon dactylon* (L.) Pers. [79]. The distribution of CMA^+^ bands between ploidies of *C. dactylon* showed an increase in the number of chromosomes with CG-rich regions as ploidy increased [79]. This trend indicates that the same number of chromosomes with consistent CMA^+^ bands positions (likely associated with NORs) is conserved in autopolyploids. Furthermore, in *Lathyrus nervosus* Lam., autotriploid and autotetraploid individuals displayed CMA/DAPI banding and a pattern of rDNA *loci* identical to those observed in diploid individuals of the same population [22]. However, further analyses are necessary in tetraploid *P. notatum* to determine whether the variation in the number of heterochromatin blocks in quartet 6 affects the functionality.

Generally, Het-C blocks negatively impact pairing and crossing-over [78,80]. Recombination suppression has been observed in several apomictic species, including *Erigeron annuus* (L.) Pers., *Tripsacum dactyloides* (L.) L., *Cenchrus ciliaris* L., *Panicum maximum* Jacq.*, Pennisetum squamulatum* Fresen, and even in *P. notatum* and *P. simplex* Morong [32,81]. Numerous studies have linked the apomixis *locus* to heterochromatin blocks and repetitive DNA regions [82,83]. Moreover, segregation distortion and suppressed recombination have been proposed to explain apomixis inheritance patterns in several plant species [81]. Martínez et al. [33,34] suggested segregation distortion for apomixis in *P. notatum* based on extensive cyto-embryological analyses, molecular markers linked to the apospory trait, and a flow cytometric seed screen. Additionally, many molecular markers in apomictic *P. notatum* segregate without recombination, indicating suppressed recombination in the genomic region controlling apospory [34,42]. Chromosomal rearrangements that suppress recombination provide a mechanistic explanation for this phenomenon in apomictic *P. notatum* [32,84]. Genetic analyses of apomixis in *Paspalum* suggested that genetic elements are located within low-recombining chromosome regions [32]. However, karyotypes of *Paspalum* species remain poorly characterized. Until more polyploid complexes are studied, the relationship between karyotype features and apomixis will remain unclear.

This study presents the first genome size estimates for *P. notatum*. Currently, the genome is considered as the chromosome complement and its DNA characteristic for an organism, and the monoploid genome size is referred to as 1Cx as a unifying term [85,86]. The genome size is sometimes measured as the sum of the total chromosome length (TLC) and expressed in micrometers (µm). Both measures demonstrated that the increment in DNA content in autotetraploid *P. notatum* was not a precise duplication of the diploid genome. Several previous studies have shown that the genome size of *Paspalum* species has been a key focus for researchers involved in germplasm characterization and breeding. Pioneering work by Sandhu et al. [87,88] developed comparative strategies based on genomic measurements of 2*x* and 4*x* bahiagrass and its hybrids. However, in both studies, ploidy indices were estimated without reporting DNA values in picograms.

The diploid cytotype exhibited a genome size of 1.41 ± 0.008 pg, while the tetraploid cytotype showed a genome size of 2.753 ± 0.015 pg. This discrepancy indicates that genome downsizing occurred during the polyploidization process, as reflected in the monoploid genome size (1Cx = 0.713 pg for diploids and 1Cx = 0.678 pg for tetraploids). This reduction in the DNA content aligns with findings in other polyploid species, where genome downsizing has been documented as a common phenomenon [89,90,91]. For instance, genome downsizing was observed in synthetic hybrids and natural tetraploids of *P. notatum* [15]. Similarly, this trend has been reported for *Paspalum* species from the *Dilatata*, *Quadrifaria*, and *Paniculata* groups, where the DNA content ranged from 1.24 pg in diploid *P. juergensii* to 3.79 pg in a hexaploid biotype of *P. dilatatum* [89].

The observed genome downsizing in tetraploid *P. notatum* also correlates with the reduction in the individual chromosome length and the total chromosome length (TCL). The tetraploid TCL did not match the expected duplication of the diploid TCL, with a reduction of approximately 4.62 µm. This suggests that structural genome changes, such as the loss of repetitive DNA sequences or chromatin, may have occurred during N genome evolution. Genome downsizing in *Paspalum* species is thought to result from mechanisms such as deletion of repetitive DNA, which helps stabilize the polyploid genome [15,89].

In diploid *P. notatum*, the monoploid genome size (1Cx) was 694.187 Mbp, according to estimates of chromosome-scale genome assembly and annotation for this species [46,47]. These findings highlight the high level of complexity and variability in genome structure across different cytotypes. Future studies should focus on integrating karyotype data with chromosome-scale assemblies to establish a more comprehensive understanding of genome organization and its implications for evolutionary adaptations.

Moreover, genome size reduction in polyploids has been associated with selective pressures to optimize cellular processes, such as DNA replication and cell division. The smaller genome size in tetraploid *P. notatum* may confer advantages in specific ecological niches by reducing the metabolic cost of maintaining a larger genome. However, additional research is needed to explore the functional consequences of genome downsizing in polyploids and its impact on traits such as stress tolerance and reproductive efficiency.

## 5. Conclusions

A deep exploration of chromosomes and karyotypes is essential for the physical detection of sequences of interest and their spatial localization on chromosomes. Chromosome karyotype studies linked to genetic sequence analysis and linkage maps provide precision tools for genetic improvement and the manipulation of advantageous and localized DNA sequences. Conversely, the identification of DNA sequences of interest can be associated with their specific chromosomal locations. However, the current challenges primarily stem from the lack of comprehensive karyotype information for numerous plant species or the description of linkage groups not yet associated with cytologically recognized chromosomes.

This study provides the first detailed report of the karyotypes of diploid and tetraploid *P. notatum*. The karyotype data presented in this study contribute to the potential integration of genomic and cytogenetic maps for *P. notatum*. Establishing an accurate and reproducible consensus for chromosome nomenclature to identify each chromosome in the diploid and tetraploid complements is a critical first step. Constitutive heterochromatin (Het-C) regions were identified for the first time in *P. notatum*. The karyotypes, heterochromatin patterns, and genome sizes support the autopolyploid origin of *P. notatum* and suggest only minor structural changes in the N genome following polyploidization.

With the advancement of technologies, such as chromosomal microdissection, flow cytometry chromosome sorting, and the association of sequences with specific chromosomal territories and landmarks, the karyotype parameters established in this study could facilitate comparative analyses of karyotype structure and evolution in *Paspalum*. These insights may also help elucidate their correlation with apomixis expression, thereby supporting future research and applications in genetic improvement programs.

## Figures and Tables

**Table 1:** List of the studied *P. notatum* accessions, ploidy level (2*n*), collection localities, and herbarium vouchers.

Species Variety	2*n*	Locality and Voucher
*P. notatum* Flüggé var. *saurae* Parodi	20	Argentina, Santa Fe, Ruta provincial 19, Colastiné, near to Tunel subfluvial. 28 March 2010. S 31°39′59.3″ W 60°35′10.4″ *Honfi & Daviña 1453* #1, #2 (MNES).
	20	Paraguay, Itapúa, Encarnación. 11 March 2015. *Honfi 1740* #2 (MNES). S 27°19′12.166″, W 55°51′52.198″
	20	Argentina, Entre Ríos, Gualeguaychú, Cerro del Indio. 9 April 2015. *Honfi 1961* #11 (MNES). S 33°4′9.199″, W 58°26′28.399″
*P. notatum* Flüggé var. *notatum*	40	Argentina, Santa Fe, surroundings access to the subfluvial tunnel. 30 September 2006. *Honfi & Daviña 1304* (MNES).
	40	Argentina, Misiones, Departamento Capital, Posadas. 3 April 2012. *Honfi 1603* #1, #2, #3 (MNES).

**Table 2:** Karyomorphometry of diploid cytotype of *P. notatum*.

Pair	*s* (µm) ± SE	*l* (µm) ± SE	*c* (µm) ± SE	*i*	Type	% HTCL
1	1.028 ± 0.066	1.181 ± 0.094	2.209 ± 0.16	0.4654	*m*	12.88
2	0.867 ± 0.075	1.086 ± 0.082	1.961 ± 0.15	0.4421	*m*	11.44
3	0.809 ± 0.063	1.057 ± 0.067	1.866 ± 0.13	0.4335	*m*	10.88
4	0.751 ± 0.053	0.998 ± 0.079	1.749 ± 0.12	0.4294	*m*	10.20
5	0.736 ± 0.042	0.925 ± 0.057	1.661 ± 0.096	0.4431	*m*	9.69
6	0.656 ± 0.042	0.874 ± 0.057	1.516 ± 0.109	0.4327	*m*	8.84
7	0.685 ± 0.059	0.787 ± 0.058	1.473 ± 0.115	0.465	*m*	8.59
8	0.656 ± 0.063	0.78 ± 0.046	1.437 ± 0.108	0.4565	*m*	8.38
9	0.598 ± 0.037	0.729 ± 0.066	1.327 ± 0.103	0.4506	*m*	7.74
10	0.671 ± 0.045	1.262 ± 0.116	1.94 ± 0.158	0.3459	*sm*	11.32

Ref. *s*: short arm, *l*: long arm, *c*: total chromosome length, SE: standard error, *i*: centromeric index, chromosome type: *m*, metacentric; *sm*, submetacentric (according to Levan et al. [48]); HTCL: haploid total chromosome length.

**Table 3:** Karyomorphometry of the autotetraploid of *P. notatum*.

Chrom	*s* (µm) ± SE	*l* (µm) ± SE	*c* (µm) ± SE	*i*	Type	% TCL
Q1-C1	1.047 ± 0.11	1.392 ± 0.04	2.411 ± 0.11	0.434	*m*	3.77
Q1-C2	1.025 ± 0.06	1.297 ± 0.12	2.323 ± 0.14	0.441	*m*	3.63
Q1-C3	0.942 ± 0.08	1.240 ± 0.11	2.180 ± 0.16	0.432	*m*	3.41
Q1-C4	0.937 ± 0.10	1.113 ± 0.03	2.051 ± 0.12	0.457	*m*	3.20
Q2-C5	0.839 ± 0.04	1.087 ± 0.10	1.975 ± 0.12	0.425	*m*	3.08
Q2-C6	0.856 ± 0.09	1.033 ± 0.05	1.889 ± 0.12	0.453	*m*	2.95
Q2-C7	0.806 ± 0.07	1.054 ± 0.07	1.860 ± 0.12	0.433	*m*	2.91
Q2-C8	0.835 ± 0.07	0.946 ± 0.06	1.813 ± 0.10	0.461	*m*	2.83
Q3-C9	0.699 ± 0.03	1.0255 ± 0.13	1.753 ± 0.09	0.399	*m*	2.74
Q3-C10	0.720 ± 0.06	0.944 ± 0.06	1.664 ± 0.11	0.433	*m*	2.60
Q3-C11	0.751 ± 0.05	0.884 ± 0.07	1.636 ± 0.11	0.459	*m*	2.55
Q3-C12	0.696 ± 0.05	0.939 ± 0.08	1.636 ± 0.11	0.426	*m*	2.55
Q4-C13	0.775 ± 0.06	0.861 ± 0.05	1.636 ± 0.11	0.474	*m*	2.55
Q4-C14	0.723 ± 0.04	0.913 ± 0.08	1.636 ± 0.11	0.442	*m*	2.55
Q4-C15	0.706 ± 0.06	0.913 ± 0.08	1.619 ± 0.13	0.436	*m*	2.53
Q4-C16	0.746± 0.06	0.872 ± 0.09	1.590 ± 0.12	0.469	*m*	2.48
Q5-C17	0.699 ± 0.03	0.838 ± 0.07	1.561 ± 0.11	0.448	*m*	2.44
Q5-C18	0.694 ± 0.05	0.832 ± 0.08	1.526 ± 0.11	0.455	*m*	2.38
Q5-C19	0.667 ± 0.05	0.835 ± 0.07	1.502 ± 0.10	0.444	*m*	2.35
Q5-C20	0.723 ± 0.06	0.780 ± 0.05	1.502 ± 0.10	0.481	*m*	2.35
Q6-C21	0.694 ± 0.05	0.837 ± 0.05	1.502 ± 0.10	0.462	*m*	2.35
Q6-C22	0.639 ± 0.03	0.772 ± 0.07	1.411 ± 0.10	0.453	*m*	2.20
Q6-C23	0.639 ± 0.03	0.772 ± 0.07	1.411 ± 0.10	0.453	*m*	2.20
Q6-C24	0.639 ± 0.03	0.772 ± 0.07	1.411 ± 0.10	0.453	*m*	2.20
Q7-C25	0.663 ± 0.05	0.720 ± 0.06	1.383 ± 0.09	0.479	*m*	2.16
Q7-C26	0.605 ± 0.07	0.777 ± 0.05	1.371 ± 0.10	0.442	*m*	2.14
Q7-C27	0.627 ± 0.04	0.720 ± 0.06	1.348 ± 0.08	0.466	*m*	2.10
Q7-C28	0.610 ± 0.05	0.720 ± 0.06	1.330 ± 0.10	0.459	*m*	2.08
Q8-C29	0.582 ± 0.05	0.748 ± 0.06	1.330 ± 0.10	0.437	*m*	2.08
Q8-C30	0.569 ± 0.05	0.705± 0.05	1.275 ± 0.09	0.447	*m*	1.99
Q8-C31	0.527 ± 0.03	0.7440 ± 0.07	1.247 ± 0.09	0.423	*m*	1.95
Q8-C32	0.550 ± 0.05	0.696 ± 0.05	1.247 ± 0.09	0.442	*m*	1.95
Q9-C33	0.522 ± 0.05	0.753 ± 0.08	1.218 ± 0.09	0.429	*m*	1.90
Q9-C34	0.522 ± 0.05	0.636 ± 0.05	1.158 ± 0.09	0.451	*m*	1.81
Q9-C35	0.4985 ± 0.04	0.579 ± 0.06	1.077 ± 0.08	0.462	*m*	1.68
Q9-C36	0.517 ± 0.03	0.560 ± 0.06	1.077 ± 0.08	0.480	*m*	1.68
Q10-C37	0.655 ± 0.08	1.301 ± 0.10	2.024 ± 0.24	0.324	*sm*	3.16
Q10-C38	0.627 ± 0.08	1.344 ± 0.14	1.971 ± 0.22	0.318	*sm*	3.08
Q10-C39	0.556 ± 0.05	1.187 ± 0.08	1.738 ± 0.13	0.320	*sm*	2.71
Q10-C40	0.550 ± 0.05	1.013 ± 0.15	1.621 ± 0.16	0.340	*sm*	2.53

Abbreviations: *s*, short arm; *l*, long arm; *c*, total chromosome length; SE, standard error; *i*, centromeric index; chromosome type: *m*, metacentric; *sm*, submetacentric (according to Levan et al. [48]); *TCL*: total chromosome length.

**Table 4:** Comparisons of genome parameters between diploid and tetraploid genetic system of *P. notatum*.

Parameter	Diploid Cytotype	Tetraploid Cytotype
2*n*	20	40
Karyotype formula	18 *m* + 2 *sm*	36 *m* + 4 *sm*
Mean chromosome length	1.714 ± 0.084 µm	1.599 ± 0.08 µm
cMin	1.3 µm	1.1 µm
cMax	2.3 µm	2.3 µm
Stebbins’s category	1A	2B
A_1_	0.22	0.22
A_2_	0.16	0.35
Chromosome with SAT	Pair 6	Quartet 6
Secondary constriction	Pair 6, short arm	Quartet 6, short arm
TCL (µm)	34.28 µm	63.94 µm
Increase in proportion of TCL (µm)	--	97.6%
Difference in TCL among cytotypes	--	4.62 µm
Difference in Het-C amount among cytotypes	−0.67%	+0.67%

Abbreviations: 2*n*, somatic chromosome number; *cMin*, minimum chromosome length; *cMax*, maximum chromosome length; *A*_1_ and *A*_2_, intra-chromosome and inter-chromosome asymmetry indexes [54], *SAT*, satellite; *TCL*, total chromosome length of the complement; *Het-C*, constitutive heterochromatin.

**Table 5:** CMA/DA/DAPI banding pattern in diploid and tetraploid *P. notatum*.

Parameters	Diploid Cytotype	Tetraploid Cytotype
Het-C bands Position	CMA + DAPI^−^ bands in distal region of short arm and satellite of pair 6. GC-rich Het-C	One chromosome with GC-rich Het-C at distal position of the short arm (CMA + DAPI^−^), and three with CMA^+^ + DAPI^0^. Satellites of quartet 6 GC-rich Het-C.
GC-rich Het-C	(+)	(+)
AT-rich Het-C	(−)	(−)
% Het-C per TCL (µm)	2.8%	3.47%
Difference in Het-C amount among cytotypes	−0.67%	+0.67%

Abbreviations: *Het-C*, constitutive heterochromatin; *TCL*, total chromosome length of the complement in micrometers (µm).

**Table 6:** Genome size of diploid and tetraploid *P. notatum*.

Sample	Ploidy	2C-Value (pg) ± SE	1Cx-Value (pg)	1Cx-Value (Mbp)
H1740 #2	2*x*	1.41 ± 0.008	0.701	685.504 × 10^9^ bp
H1961 #11	2*x*	1.438 ± 0.017	0.719	702.871 × 10^9^ bp
Mean GS	2*x*	1.424	0.713	697.314 × 10^9^ bp
H1603 #1	4*x*	2.753 ± 0.015	0.678	1321.910 × 10^9^ bp

Abbreviations: *GS*, genome size; *2C-value*: DNA content of the whole complement of chromosomes; *pg*, picograms; *SE*, standard error; *1Cx-value*: Monoploid genome size. *Mbp*, mega base pair, 1 pg = 0.978 × 10^9^ bp [58,59].

## Data Availability

The original contributions presented in the study are included in the article, further inquiries can be directed to the corresponding author.

## Abbreviations

The following abbreviations are used in this manuscript:

*TCL*	Total chromosome length of the complement
*HTCL*	Haploid total chromosome length
*i*	Centromeric Index
*r*	Arm ratio
*R*	Ratio of the shortest to the longest chromosome pair
A_1_	Intra-karyotypic asymmetry index
A_2_.	Inter-karyotypic asymmetry index
CMA	Chromomicyn A3
DA	Distamicyn A
DAPI	4′-6-diamidino-2-phenilindol
Het-C	Constitutive heterochromatin
C-DNA_abs_	Absolute DNA content
1Cx	Monoploid genome size

## References

[1] Bennett M.D., Grant W.F. (1984). The genome, the natural karyotype and biosystematics. Plant Biosystematics.

[2] Brazier T., Glémin S. (2022). Diversity and determinants of recombination landscapes in flowering plants. PLoS Genet..

[3] Johnston S.E. (2024). Understanding the Genetic Basis of Variation in Meiotic Recombination: Past, Present, and Future. Mol. Biol. Evol..

[4] Vimala Y., Lavania S., Chandra Lavania U. (2021). Chromosome change and karyotype differentiation implications in speciation and plant systematics. Nucleus.

[5] Hamon P., Siljak-Yakovlev S., Srisuwan S., Robin O., Poncet V., Hamon S., de Kochko A. (2009). Physical mapping of rDNA and heterochromatin in chromosomes of 16 *Coffea* species: A revised view of species differentiation. Chromosome Res..

[6] Ribeiro T., Barrela R.M., Bergès H., Marques C., Loureiro J., Morais-Cecílio L., Paiva J.A.P. (2016). Advancing *Eucalyptus* Genomics: Cytogenomics Reveals Conservation of *Eucalyptus* Genomes. Front. Plant Sci..

[7] Guerra M. (2000). Patterns of heterochromatin distribution in plant chromosomes. Gen. Mol. Biol..

[8] Roa F., Guerra M. (2012). Distribution of 45S rDNA sites in chromosomes of plants: Structural and evolutionary implications. BMC Evol. Biol..

[9] Gianini Aquino A.C., González Flores M., Honfi A.I., Daviña J.R. (2023). Heterochromatin patterns in four diploid *Zephyranthes* species with different basic chromosome number (Amaryllidaceae). Darwiniana.

[10] Reutemann A.V., Martínez E.J., Daviña J.R., Hojsgaard D.H., Honfi A.I. (2021). El cariotipo de *Paspalum cromyorrhizon* diploide y tetraploide (Poaceae, Panicoideae, Paspaleae). Darwiniana.

[11] Rosselló J.A., Maravilla A.J., Rosato M. (2022). The nuclear 35S rDNA world in plant systematics and evolution: A primer of cautions and common misconceptions in cytogenetic studies. Front. Plant Sci..

[12] Luo X., Liu Y., Gong X., Ye M., Xiao Q., Zeng Z. (2024). Karyotype description and comparative chromosomal mapping of 5S rDNA in 42 Species. Genes.

[13] Las Peñas M.L., Urdampilleta J.D., Bernardello G., Forni-Martins E.R. (2009). Karyotypes, heterochromatin, and physical mapping of 18S-26S rDNA in Cactaceae. Cytogenet. Genome Res..

[14] Hasterok R., Wang K., Jenkins G. (2020). Progressive refinement of the karyotyping of *Brachypodium* genomes. New Phytol..

[15] Galdeano F., Urbani M.H., Sartor M.E., Honfi A.I., Espinoza F., Quarin C.L. (2016). Relative DNA content in diploid, polyploid, and multiploid species of Paspalum (Poaceae) with relation to reproductive mode and taxonomy. J. Pl. Res..

[16] Doležel J., Urbiš P., Said M., Lucretti S., Molnár I. (2023). Flow cytometric analysis and sorting of plant chromosomes. Nucleus.

[17] Stace C.A. (1980). Plant Taxonomy and Biosystematics.

[18] Heng J., Heng H.H. (2021). Karyotype coding: The creation and maintenance of system information for complexity and biodiversity. BioSystem.

[19] Stebbins G.L. (1947). Types of polyploids: Their classification and significance. Adv. Genet..

[20] Parisod C., Holderegger R., Brochmann C. (2010). Evolutionary consequences of autopolyploidy. New Phytol..

[21] Lv Z., Nyarko C.A., Ramtekey V., Mason A.S. (2024). Defining autopolyploidy: Cytology, genetics, and taxonomy. Am. J. Bot..

[22] Chalup L., Grabiele M., Solís Neffa V., Seijo G. (2012). Structural karyotypic variability and polyploidy in natural populations of the South American *Lathyrus nervosus* Lam. (Fabaceae). Plant Syst. Evol..

[23] Reis A.C., Chester M., de Sousa S.M. (2022). Chromosomal view of *Lippia alba*, a tropical polyploid complex under genome stabilization process. Protoplasma.

[24] Giacò A., De Giorgi P., Astuti G., Varaldo L., Sáez L., Carballal R., Serrano M., Casazza G., Caputo P., Bacchetta G. (2022). Diploids and polyploids in the *Santolina chamaecyparissus* complex (Asteraceae) show different karyotype asymmetry. Plant Biosyst..

[25] Maluszynska J., Heslop-Harrison J.S. (1993). Localization of tandemly repetead DNA sequences in *Arabidopsis thaliana*. Plant J..

[26] Sochorová J., Coriton O., Kuderová A., Lunerová J., Chèvre A.M., Kovařik A. (2017). Gene conversion events and variable degree of homogenization of rDNA loci in cultivars of *Brassica napus*. Ann. Bot..

[27] Meng Z., Shi S., Shen H., Xie Q., Li H. (2023). Haplotype-specific chromosome painting provides insights into the chromosomal characteristics in self-duplicating autotetraploid sugarcane. Ind. Crop. Prod..

[28] Sumner A.T. (1990). Chromosome Banding.

[29] Barros e Silva A.E., Guerra M. (2010). The meaning of DAPI bands observed after C-banding and FISH procedures. Biotech. Histochem..

[30] Chase A. (1929). The North American species of *Paspalum*. Contr. U. S. Natl. Herb..

[31] Acuña C.A., Martínez E.J., Zilli A.L., Brugnoli E.A., Espinoza F., Marcón F., Urbani M.H., Quarin C.L. (2019). Reproductive systems in *Paspalum*: Relevance for germplasm collection and conservation, breeding techniques, and adoption of released cultivars. Front. Plant Sci..

[32] Ortiz J.P.A., Pupilli F., Acuña C.A., Leblanc O., Pessino S.C. (2020). How to become an apomixis model: The multifaceted case of *Paspalum*. Genes.

[33] Martínez E.J., Urbani M.H., Quarín C.L., Ortiz J.P.A. (2001). Inheritance of apospory in bahiagrass, *Paspalum notatum*. Hereditas.

[34] Martínez E.J., Hopp H., Stein J., Ortiz J.P.A., Quarin C.L. (2003). Genetic characterization of apospory in tetraploid *Paspalum notatum* based on the identification of linked molecular markers. Mol. Breed..

[35] D’Aurelio L.D., Espinoza F., Quarin C.L., Pessino S.C. (2004). Genetic diversity in sexual diploid and apomictic tetraploid populations of *Paspalum notatum* situated in sympatry or allopatry. Plant Syst. Evol..

[36] Burton G.W. (1955). Breeding Pensacola bahiagrass, *Paspalum notatum*: I. Method of reproduction. Agron. J..

[37] Quarin C.L., Norrmann G.A., Urbani M.H. (1989). Polyploidization in aposporous *Paspalum* species. Apomixis Newsl..

[38] Tischler C.R., Burson B.L. (1995). Evaluating different bahiagrass cytotypes for heat tolerance and leaf epicular wax content. Euphytica.

[39] Forbes I.J.R., Burton G.W. (1961). Cytology of diploids, natural and Induced tetraploids, and intra-species hybrids of Bahiagrass, *Paspalum notatum* Flügge. Crop Sci..

[40] Quarin C.L., Burson B.L., Burton G.W. (1984). Cytology of intra-and interspecific hybrids between two cytotypes of Paspalum notatum and P. cromyorrhizon. Bot. Gaz..

[41] Quarin C.L., Norrmann G.A. (1987). Cytology and reproductive behavior of *Paspalum equitans, P. ionanthum*, and their hybrids with diploid and tetraploid cytotypes of *P. cromyorrhizon*. Bot. Gaz..

[42] Stein J., Quarin C.L., Martnez E.J., Pessino S.C., Ortiz J.P.A. (2004). Tetraploid races of *Paspalum notatum* show polysomic inheritance and preferential chromosome pairing around the apospory-controlling locus. Theor. Appl. Genet..

[43] Stein J., Pessino S.C., Martínez E.J., Pıa M., Rodriguez L., Siena A., Quarin C.L., Ortiz J.P.A. (2007). A genetic map of tetraploid *Paspalum notatum* Fluggé (bahiagrass) based on single-dose molecular markers. Mol. Breed..

[44] Ortiz J.P.A., Pessino S.C., Bhat V., Hayward M.D., Quarin C.L. (2001). A genetic linkage map of diploid *Paspalum notatum*. Crop Sci..

[45] Quarin C.L., Espinoza F., Martínez E.J., Pessino S.C., Bovo O.A. (2001). A rise of ploidy level induces the expression of apomixis in *Paspalum notatum*. Sex Plant Reprod..

[46] Yan Z., Yan Z., Cong L., Liu H., Chen Y., Sun J., Ma L., Wang A., Miao F., Song H. (2022). High-quality chromosome-scale de novo assembly of the *Paspalum notatum* ‘Flugge’ genome. BMC Genom..

[47] Vega J.M., Podio M., Orjuela J., Siena L.A., Pessino S.C., Combes M.C., Mariac C., Albertini E., Pupilli F., Ortiz J.P.A. (2024). Chromosome-scale genome assembly and annotation of *Paspalum notatum* Flüggé var. saurae. Sci. Data.

[48] Levan A., Fredga K., Sandberg A.A. (1964). Nomenclature for centromeric position on chromosomes. Hereditas.

[49] Reeves A. (2001). MicroMeasure: A new computer program for the collection and analysis of cytogenetic data. Genome.

[50] Honfi A.I., Bolzán A.D., Daviña J.R. (2017). Dimensión Cromosómica. Cienc. Investig. AAPC.

[51] Battaglia E. (1955). Chromosome morphology and terminology. Caryologia.

[52] Battaglia E. (1999). The chromosome satellite (Navashin’s Sputnik or Satelles): A terminological comment. Acta Biol. Cracov. Ser. Bot..

[53] Stebbins G.L. (1971). Chromosomal Evolution in Higher Plants.

[54] Romero Zarco C. (1986). A new method for estimating karyotype asymmetry. Taxon.

[55] Schwarzacher T., Ambros P., Schweizer D. (1980). Application of Giemsa banding to orchid karyotype analysis. Plant Syst. Evol..

[56] Daviña J.R., Gianini Aquino A.C., Rodríguez Mata O.A., Tapia Campos E., Barba-Gonzalez R., Honfi A.I. (2022). Chromosomic studies in *Zephyranthes citrina* Baker (Amaryllidaceae), a polyploid ornamental. BAG J. Basic Appl. Genet..

[57] Schweizer D. (1976). Reverse fluorescent chromosome banding with chromomycin and DAPI. Chromosoma.

[58] Dolezel J., Bartos J., Voglmayr H., Greilhuber J. (2003). Nuclear DNA content and genome size of trout and human. Cytometry.

[59] Dolezel J., Greilhuber J., Suda J. (2007). Estimation of nuclear DNA content in plants using flow cytometry. Nat. Protoc..

[60] Pozzobon M.T., Valls J.F.M. (1997). Chromosome number in germplasm accessions of *Paspalum notatum* (Gramineae). Braz. J. Genet..

[61] Gates R.N., Quarin C.L., Pedreira C.G.S., Moser L.E., Burson B.L., Sollenberger L.E. (2004). Bahiagrass, in Warm-Season (C4) Grasses.

[62] Hojsgaard D.H., Honfi A.I., Rua G., Daviña J.R. (2009). Chromosome numbers and ploidy levels of *Paspalum* species from subtropical South America (Poaceae) *Genet*. Resour. Crop Evol..

[63] Reutemann A.V., Rua G.H., Daviña J.R., Honfi A.I. (2019). IAPT chromosome data 31/11. In Marhold, K. & Kucera, J. (eds.) & al., IAPT chromosome data 31. Taxon.

[64] Burson B.L. (1981). Cytogenetic relationships between *Paspalum jurgensii* and *P. intermedium*, *P. vaginatum*, and *P. setaceum* var. *ciliatifolium*. Crop Sci..

[65] Quarin C.L., Burson B.L. (1983). Cytogenetic relations among *Paspalum notatum* var. *saurae*, *P. pumilum*, *P. indecorum*, and *P. vaginatum*. Bot. Gaz..

[66] Bonilla J., Quarin C.L. (1997). Diplosporous and aposporous apomixis in a pentaploid race of *Paspalum minus*. Plant Sci..

[67] Reutemann A.V., Martínez E.J., Schedler M., Daviña J.R., Hojsgaard D.H., Honfi A.I. (2022). Uniparentality: Advantages for range expansion in diploid and diploid-autopolyploid species. Bot. J. Linn. Soc..

[68] Schedler M., Reutemann A.V., Hojsgaard D.H., Zilli A.L., Brugnoli E.A., Galdeano F., Acuña C.A., Honfi A.I., Martínez E.J. (2023). Alternative evolutionary pathways in *Paspalum* involving allotetraploidy, sexuality, and varied mating systems. Genes.

[69] Zuloaga F.O., Morrone O. (2005). Revisión de las especies de *Paspalum* para América del Sur austral (Argentina, Bolivia, sur del Brasil, Chile, Paraguay y Uruguay). Ann. Mo. Bot. Gard. Monogr. Syst. Bot..

[70] Ortiz J.P.A., Quarin C.L., Pessino S.C., Acuna C., Martínez E.J., Espinoza F., Pupilli F. (2013). Harnessing apomictic reproduction in grasses: What we have learned from *Paspalum*. Ann. Bot..

[71] Peruzzi L., Leitch I.J., Caparelli K.F. (2009). Chromosome diversity and evolution in Liliaceae. Ann. Bot..

[72] Speranza P., Vaio M., Mazzella C. (2003). Karyotypes of two cytotypes of *Paspalum quadrifarium* Lam. (Poaceae): An alternative technique for small chromosomes in plants. Genet. Mol. Biol..

[73] Honfi A.I., Morrone O., Zuloaga F.O. (2021). Chromosome numbers and ploidy levels of some Paniceae and Paspaleae species (Poaceae, Panicoideae). Ann. Mo. Bot. Gard..

[74] Wachtler F., Stahl A. (1993). The nucleolus: A structural and functional interpretation. Micron.

[75] Vaio M., Speranza P., Valls J.F., Guerra M., Mazzella C. (2005). Localization of the 5S and 45S rDNA Sites and cpDNA Sequence Analysis in Species of the Quadrifaria Group of *Paspalum* (Poaceae, Paniceae). Ann. Bot..

[76] Vaio M., Mazzella C., Guerra M., Speranza P. (2019). Effects of the diploidisation process upon the 5S and 35S rDNA sequences in the allopolyploid species of the Dilatata group of *Paspalum* (Poaceae, Paniceae). Aust. J. Bot..

[77] Greilhuber J. (1982). Trends in der chromosomen evolution von *Scilla* (Liliaceae). Stapfia.

[78] John B., Verma R.S. (1988). The biology of heterochromatin. Heterochromatin, Molecular and Structural Aspects.

[79] Arantes Chaves A.L., Bezerra Chiavegatto R., Gandolfi Benites F.R., Techio V.H. (2019). Comparative karyotype analysis among cytotypes of *Cynodon dactylon* (L.) Pers. (Poaceae). Mol. Biol. Rep..

[80] John B. (1990). Meiosis.

[81] Ozias-Akins P., van Dijk P.J. (2007). Mendelian Genetics of Apomixis in Plants. Annu. Rev. Genet..

[82] Calderini O., Chang S.B., de Jong H., Busti A., Paolocci F., Arcioni S., de Vries S.C., Abma-Henkens M.H.C., Lankhorst R.M.K., Donnison L.S. (2006). Molecular cytogenetics and DNA sequence analysis of an apomixis-linked BAC in *Paspalum simplex* reveal a non pericentromere location and partial microcolinearity with rice. Theor. Appl. Genet..

[83] Conner J.A., Goel S., Gunawan G., Cordonnier-Pratt M.M., Johnson V.E., Liang C., Wang H., Pratt L.H., Mullet J.E., DeBarry J. (2008). Sequence analysis of bacterial artificial chromosome clones from the apospory-specific genomic region of *Pennisetum* and *Cenchrus*. Plant Physiol..

[84] Podio M., Siena L.A., Hojsgaard D., Stein J., Quarin C.L., Ortiz J.P.A. (2012). Evaluation of meiotic abnormalities and pollen viability in aposporous and sexual tetraploid *Paspalum notatum* (Poaceae). Plant Syst. Evol..

[85] Bennett M.D., Leitch Y. (2005). Plant Genome Size Research: A Field In Focus. Ann. Bot..

[86] Greilhuber J., Dolezel J., Lysák M.A., Bennett M.D. (2005). The origin, evolution and proposed stabilization of the terms ’genome size’ and ’C-value’ to describe nuclear DNA contents. Ann. Bot..

[87] Sandhu S., James V.A., Quesenberry K.H.., Altpeter F. (2009). Risk assessment of transgenic apomictic tetraploid *bahiagrass*, cytogenetics, breeding behavior and performance of intra-specific hybrids. Theor. Appl. Genet..

[88] Sandhu S., Blount A.R., Quesenberry K.H., Altpeter F. (2010). Apomixis and ploidy barrier suppress pollen-mediated gene flow in field grown transgenic turf and forage grass (*Paspalum notatum* Flüggé). Theor. Appl. Genet..

[89] Vaio M., Mazzella C., Porro V., Speranza P., López-Carro B., Estramil E., Folle G.A. (2007). Nuclear DNA content in allo-polyploid species and synthetic hybrids in the grass genus Paspalum. Pl. Syst. Evol..

[90] Maretti Gonçalves T., Gomes Ferreira J.R., Baccili Zanotto Vigna B., Sousa Azevedo A.L., Toniolo Pozzobon M., Pereira Fávero A. (2021). Reproductive mode and DNA content of *Paspalum* accessions from Plicatula group. Reproduction and DNA content of *Paspalum*. Flora.

[91] Matta F.P., Fávero A.P., Zanotto Vigna B.B., Mattos Cavallari M., Alves F., Oliveira F.A., Pereira de Souza A., Pozzobon M.T., Sousa Azevedo A.L., Gusmão M.R. (2024). Characterization of *Paspalum* genotypes for turfgrass cultivars development. Crop Sci..

